# The Performance of a Self-Flocculating Microalga *Chlorococcum* sp. GD in Wastewater with Different Ammonia Concentrations

**DOI:** 10.3390/ijerph15030434

**Published:** 2018-03-02

**Authors:** Junping Lv, Xuechun Wang, Wei Liu, Jia Feng, Qi Liu, Fangru Nan, Xiaoyan Jiao, Shulian Xie

**Affiliations:** 1 School of Life Science, Shanxi University, Taiyuan 030006, China; lvjunping024@sxu.edu.cn (J.L.); 201623101009@email.sxu.edu.cn (X.W.); 201523103002@email.sxu.edu.cn (W.L.); fengj@sxu.edu.cn (J.F.); liuqi@sxu.edu.cn (Q.L.); Nangfr@sxu.edu.cn (F.N.); 2 Institute of Agricultural Environment and Resource, Shanxi Academy of Agricultural Sciences, Taiyuan 030031, China; jiaoxiaoyan@sxagri.ac.cn

**Keywords:** ammonia nitrogen, biomass production, lipid accumulation, wastewater treatment, self-flocculation

## Abstract

The performance of a self-flocculating microalga *Chlorococcum* sp. GD on the flocculation, growth, and lipid accumulation in wastewater with different ammonia nitrogen concentrations was investigated. It was revealed that relative high ammonia nitrogen concentration (20–50 mg·L^−1^) was beneficial to the flocculation of *Chlorococcum* sp. GD, and the highest flocculating efficiency was up to 84.4%. It was also found that the highest flocculating efficiency occurred in the middle of the culture (4–5 days) regardless of initial ammonia concentration in wastewater. It was speculated that high flocculating efficiency was likely related to the production of extracellular proteins. 20 mg·L^−1^ of ammonia was found to be a preferred concentration for both biomass production and lipid accumulation. 92.8% COD, 98.8% ammonia, and 69.4% phosphorus were removed when *Chlorococcum* sp. GD was cultivated in wastewater with 20 mg·L^−1^ ammonia. The novelty and significance of the investigation was the integration of flocculation, biomass production, wastewater treatment, and lipid accumulation, simultaneously, which made *Chlorococcum* sp. GD a potential candidate for wastewater treatment and biodiesel production if harvested in wastewater with suitable ammonia nitrogen concentration.

## 1. Introduction

The integration of microalgae-based wastewater treatment and lipid production has major advantages for both industries [1,2]. Nevertheless, there are some challenges on microalgal harvesting from wastewater due to small cell size, low cell density, and homogeneous distribution of cell in culture systems [3]. The contribution of the harvesting cost to the total cost is reportedly in the range of 20–30% or beyond [4]. Therefore, improving the efficiency of microalgal harvesting is very important for the process of microalgae-based wastewater treatment and lipid production.

Currently, microalgal harvesting mainly involves physical-, chemical-, and biological-based methods. For example, efficiencies higher than 90% are reached by applying ultrasound waves to harvest microalgae [5]. Xu et al. [6] reports that chitosan is an effective flocculant for concentrating *Chlorella sorokiniana* with more than 99% efficiency. Bioflocculant production from *Solibacillus silvestris* W01 shows 90% flocculating efficiency on *Nannochloropsis oceanica* [7]. Some other flocculants, such as aluminum nitrate sulfate, cationic starch and poly γ-glutamic acid also exhibit excellent flocculating efficiency (>79%) on microalgae [8]. However, high energy consumption, production cost, and potential biological toxicity become a bottleneck for the further application of above harvesting technologies.

Self-flocculating microalgae are a kind of microalgae that can aggregate together by themselves. The aggregate can be easily harvested by sedimentation. Currently, some self-flocculating microalgae, such as *Ankistrodesmus falcatus*, *C*. *vulgaris* JSC-7, *Ettlia texensis*, *Scenedesmus obliquus* AS-6-1, *Scenedesmus* sp. BH, and *Tetraselmis suecica*, have been screened [9,10,11,12,13,14,15]. It has been reported that the flocculating efficiency of *Scenedesmus* sp. BH is up to 92.3% [14]. Because the self-flocculating process of microalgae is a spontaneous behavior, it does not incur extra cost for microalgal harvesting compared to the high cost of separation and purification of flocculants from microorganisms for biological flocculation [16]. Compared to physical and chemical flocculation [17], there is no biological toxicity and almost no energy consumption in the self-flocculating process. The flocculating efficiency of self-flocculating microalgae is also comparable to that of traditional flocculation technologies. Therefore, flocculation technology based on cultivation of self-flocculating microalgae exhibits some potential advantages of high efficiency, environment-friendliness, and low cost; it represents the future research direction of microalgal harvesting.

For microalgal cultivation in wastewaters, ammonia is one important form of nitrogen and can influence microalgal growth and nutrient uptake [18,19]. The response of flocculating capability to ammonia concentration is worthy of attention for the cultivation of self-flocculating microalgae. It is hoped that self-flocculating microalgae can maintain excellent flocculating ability to facilitate the efficient recovery of cells from wastewater, in addition to keeping the high growth potential and nutrient uptake efficiency.

At present, the flocculating ability of self-flocculating microalgae under different growth phasees, temperatures, and pHs has been investigated. It has been demonstrated that the suitable growth phase, temperature, and pH are beneficial to flocculation of self-flocculating microalgae [12,20]. Nevertheless, the flocculating property of self-flocculating microalgae at different ammonia concentrations is not clear, and there are no papers published so far. The effect of ammonia nitrogen on the growth of self-flocculating microalgae is also unknown.

*Chlorococcum* sp. GD belonging to green microalgae has been reported to exhibit excellent self-flocculating capability in secondary effluent [21], which was comparable to that of *C*. *vulgaris* JSC-7, *E*. *texensis*, and *S*. *obliquus* AS-6-1 [11,12,13]. In the present study, the performance of *Chlorococcum* sp. GD on the flocculation with different ammonia nitrogen concentrations is investigated. The response of growth to varied ammonia concentrations is also analyzed. The novelty and significance of the study is to evaluate the possibility of the integration of sewage treatment, biomass production, and microalgal harvesting of *Chlorococcum* sp. GD, simultaneously, by altering ammonia nitrogen concentration in wastewater.

## 2. Materials and Methods

### 2.1. Microalgal Strain

A strain of *Chlorococcum* sp. GD was used in the study. It was isolated from the moss *Entodon obtusatus* of Shanxi Province, China [22] and exhibited excellent self-flocculating property as described by Lv et al. [21].

### 2.2. Synthetic Wastewater

In the present study, synthetic wastewater was formulated to simulate municipal wastewater. The exact concentration of COD and phosphorus of the synthetic wastewater was derived from the average concentration of municipal wastewater in Shanxi Province, China. The composition of macro element of the synthetic wastewater was provided by Aslan and Kapdan [23] with some modifications. The composition and concentration of trace elements in the synthetic wastewater was consistent to those in Blue-Green Medium (BG11). The exact compositions of the synthetic wastewater were as follows (mg/L): glucose 350, NH_4_Cl 38.21–191.04, KH_2_PO_4_ 21.97, NaHCO_3_ 100, NaCl 64, MgSO_4_ 90, FeSO_4_ 5, CaCl_2_ 25, H_3_BO_3_ 2.86, MnCl_2_·4H_2_O 1.86, ZnSO_4_·7H_2_O 0.22, Na_2_MoO_4_·2H_2_O 0.39, CuSO_4_·5H_2_O 0.08, and Co(NO_3_)_2_·6H_2_O 0.05. The COD of the synthetic wastewater was around 325 mg·L^−1^ and total phosphorus concentration was 5 mg·L^−1^. The ammonia concentration varied from 10 to 50 mg·L^−1^. In the study, synthetic wastewater was sterilized (121 °C for 20 min) before experiments.

### 2.3. Experimental Set-Up

In the study, *Chlorococcum* sp. GD was firstly cultivated in BG11 for 13 days. After that, microalgal suspension was centrifuged at 5000 rpm for 5 min. The pellet was then washed with deionized water and centrifuged at 5000 rpm for 5 min again. The washed pellet was distributed in synthetic wastewater and inoculated into 500 mL transparent conical flasks with an initial concentration of 20 mg·L^−1^. In order to investigate the effect of different ammonia nitrogen concentrations, four samples with initial ammonia nitrogen of 10, 20, 30, and 50 mg·L^−1^ were prepared. Cultures were grown at a constant temperature of 25 °C in a shaker with a shaking rate of 160 revolutions per minute (rpm). The cultures were illuminated with fluorescent lamps, providing an incident light intensity of 3000 lux under 14 h:10 h light:dark cycle. After daily sampling, pH of cultures was adjusted to 7.5 by supplementing HCl or NaOH solution. All these experiments were performed in triplicate.

### 2.4. Analytical Methods

#### 2.4.1. Flocculating Ability Test

During the process of microalgal cultivation, 25 mL culture was harvested and distributed in 25 mL cylindrical glass tubes. The culture was gently mixed for 1 min at room temperature. After that, an aliquot of the culture was withdrawn at a height of two-thirds from the bottle when the culture had settled for 3 h. The optical density of the aliquots was then measured at 680 nm. The flocculating ability was calculated using the following Equation (1):

(1) $$\mathrm{Flocculating}\;\mathrm{ability}=\frac{A-B}{A}\times 100\%$$

in which A and B are the optical density (OD_680_) of the aliquot before and after flocculation.

#### 2.4.2. Extraction and Analysis of Extracellular Polymeric Substances (EPS)

EPS mainly consisted of proteins and polysaccharides. The EPS were extracted according to procedures of Yang and Li [24] with some modifications. Microalgal suspension was dewatered by centrifugation at 5000 rpm for 5 min. The pellet was washed with deionized water and centrifuged at 5000 rpm for 5 min again. The washed pellet was diluted with deionized water and was heated to 80 °C for 30 min. The mixture was then centrifuged at 10,000 rpm for 10 min. After that, the supernatant was filtered with 0.45 μm acetate cellulose membranes, and the filtrate was regarded as EPS fraction. Protein concentration was analyzed by the coomassic brilliant blue method [25] using albumin from bovine serum (BSA) as the standard. Polysaccharides concentration was analyzed by the Anthrone method [26] with glucose as the standard.

#### 2.4.3. Determination of Microalgal Growth

Microalgal biomass concentration was measured according to the method of suspended solid (SS) measurement [27]. The specific growth rate, mean microalgal biomass production, and double time during the cultivation period were calculated by Equations (2)–(4).

(2) $$\mathrm{Specific}\;\mathrm{growth}\;\mathrm{rate}\;(\mu ,d^{-1})=\ln({\mathrm{DW}}_{t2}-{\mathrm{DW}}_{t1})/(t_{2}-t_{1})$$

(3) $$\mathrm{Mean}\;\mathrm{biomass}\;\mathrm{productivity}\;(\mathrm{mg}DW\cdot L^{-1}\cdot d^{-1})=({\mathrm{DW}}_{t2}-{\mathrm{DW}}_{t1})/t$$

(4) Double time (day)=ln2/μ

In Equations (2) and (3), ${\mathrm{DW}}_{t1}$ and ${\mathrm{DW}}_{t2}$ represent the microalgal dry weight on day t_1_ and t_2_, mg·L^−1^; t_1_ and t_2_ are the cultivation time, d. 

#### 2.4.4. Determination of Water Quality

For the measurement of water quality, the microalgal suspension was centrifuged at 6000 rpm for 5 min, and the supernatant was filtered through 0.22 μm membranes. The filtered supernatant was then used for the determination of chemical oxygen demand (COD), ammonia nitrogen, and total phosphorous. COD, ammonia, and total phosphorus were analyzed by dichromate method, Nessler’s reagent spectrophotometry, and ammonium molybdate spectrophotometric method, respectively [27].

Michaelis–Menten kinetic can be used to evaluate the ammonia nitrogen removal capability of microalgae [23].

(5) $${R=R}_{\max}\times {S/(K}_{m}+S)$$

in which R is the ammonia nitrogen removal rate, R_max_ is the maximal ammonia nitrogen removal rate, S is the ammonia nitrogen concentration, and K_m_ is the ammonia nitrogen concentration at which ammonia removal rate reaches half-maximum.

Equation (5) can be linearized in double reciprocal form as in Equation (6) to determine the ammonia nitrogen removal kinetic coefficients K_m_ and V_max_ [23].

(6) $$1/R_{X_{i}}{=1/V}_{\max}{+K}_{m}{/(V}_{\max}\times S_{0})$$

in which $R_{X_{i}}$ (mg N·g^−1^ DW·d^−1^) is the specific ammonia nitrogen removal rate per unit microalgal biomass; S_0_ (mg·L^−1^) is the initial ammonia nitrogen concentration; and V_max_ (mg N·g^−1^ DW·d^−1^) is the maximal ammonia nitrogen removal rate.

#### 2.4.5. Determination of Microalgal Lipid

During the cultivation of microalgae, a suspension of microalgae (240 μL) was stained with 0.5 mg·mL^−1^ Nile red (9-diethylamino-5H-benzo(α)phenoxazine-5-one, dissolved in DMSO, 1 μL), and the mixture was incubated for 10 min at 37 °C. The fluorescence of the mixture was then determined using a microplate reader Tecan Infinite 200 Pro (Tecan, Switzerland) with a 96-well plate. The fluorescence of microalgae alone was measured. The fluorescence of Nile red alone was also measured. The fluorescence intensity of microalgal lipid was obtained when the autofluorescence of microalgae and Nile red was subtracted. The excitation and emission wavelengths were 543 and 598 nm, respectively. Each experiment was performed in triplicate.

The specific Nile Red fluorescence intensity was calculated as following:

(7) $$\mathrm{sFI}=\mathrm{FI}\times {\mathrm{DW}}^{-1}$$

in which sFI is the specific fluorescence intensity (a.u.·mg^−1^ DW biomass), FI is the total Nile Red fluorescence intensity of 240 μL microalgal suspension (a.u.), and DW is biomass dry weight in 240 μL microalgal suspension.

At the end of the culture, the total lipid was extracted with a chloroform/methanol solution (1/1, *v*/*v*) and was quantified gravimetrically [28].

#### 2.4.6. Statistical Analysis

The measured value was expressed as the mean ± standard deviation. If necessary, data were analyzed by analysis of variance (ANOVA) conducting by Statistical Product and Service Solutions (SPSS) software (version 19.0, IBM Corporation, Armonk, NY, USA). There was a statistically significant difference when *p* < 0.05. Pearson correlation analysis was also conducted by SPSS software (version 19.0), and there was a significant relationship when *p* < 0.05.

## 3. Results

### 3.1. The Flocculating Property of Chlorococcum sp. GD in Wastewater with Different Ammonia Nitrogen Concentrations

**Figure 1:**
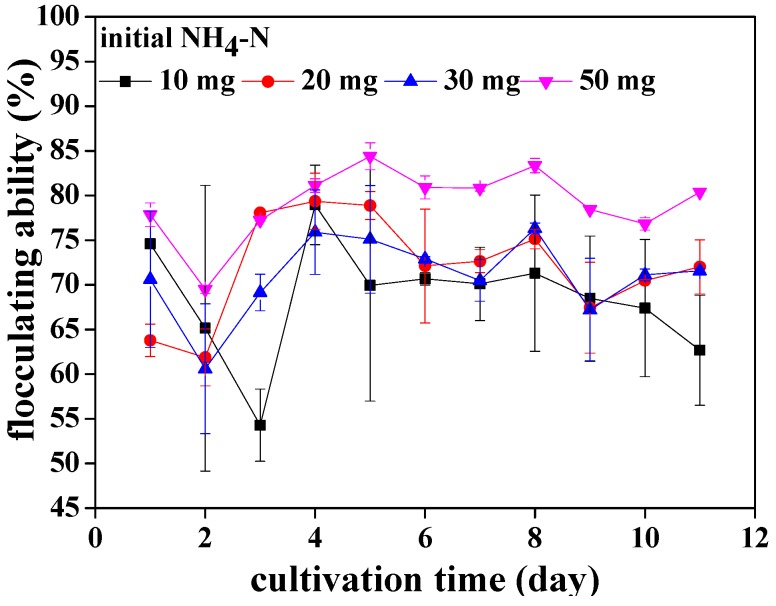
The flocculating ability of *Chlorococcum* sp. GD for 3 h settling under different ammonia nitrogen concentrations.

In Figure 1, the flocculating ability of *Chlorococcum* sp. GD cultivated with different ammonia nitrogen concentrations is presented. Throughout the culture period, the flocculating ability of *Chlorococcum* sp. GD ranged from 54.3% to 84.4%. More specifically, the flocculating ability of *Chlorococcum* sp. GD cultivated with 10 mg·L^−1^ ammonia nitrogen decreased sharply from 74.6% to 54.3% after 3 days of culture. It then increased to 79% on the 4th day. Afterwards, flocculation efficiency gradually decreased to 62.7%. For *Chlorococcum* sp. GD cultivated with 20 mg·L^−1^ ammonia nitrogen, the highest flocculating ability was 79.4% on the 4th day. Then, the flocculating ability slightly decreased and maintained at around 72% until the end of cultivation. For *Chlorococcum* sp. GD cultivated with 30 mg·L^−1^ ammonia nitrogen, the flocculating ability remained at about 70%, except for a significant decrease to 60.6% on the 2nd day. The flocculating ability of *Chlorococcum* sp. GD cultivated with 50 mg·L^−1^ ammonia nitrogen was highest and maintained at about 80% at the end of the culture. Generally speaking, the flocculating ability of *Chlorococcum* sp. GD was affected by ammonia nitrogen concentration in wastewater. *Chlorococcum* sp. GD had an excellent and stable flocculating performance when cultivated in wastewater with 50 mg·L^−1^ ammonia nitrogen. *Chlorococcum* sp. GD cultivated with 10 mg·L^−1^ ammonia nitrogen exhibited the worst flocculation ability. It was also found that the flocculating ability of *Chlorococcum* sp. GD was dynamic when cultivated in wastewater with different ammonia nitrogen concentration. The highest flocculating efficiency occurred in the middle of the culture (4–5 days) regardless of initial ammonia nitrogen concentration in wastewater.

**Figure 2:**
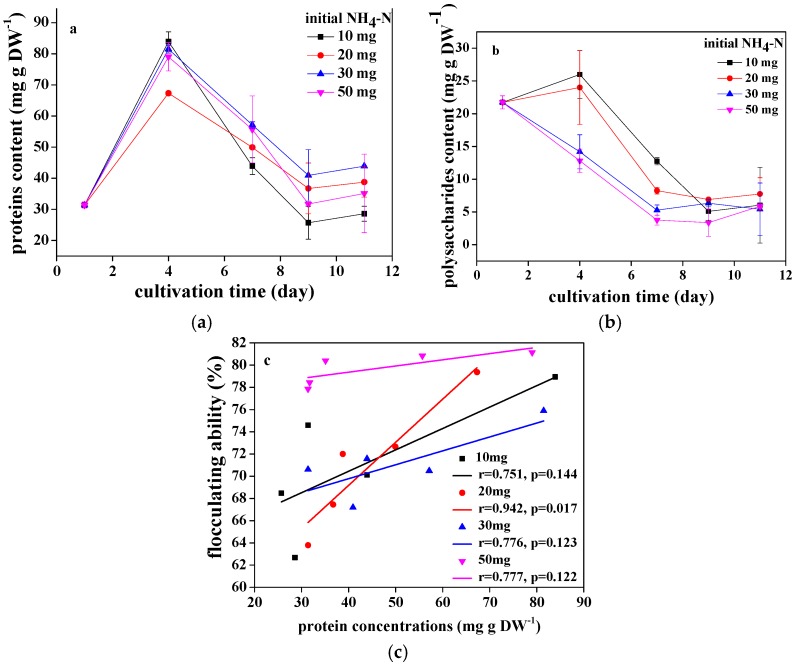
The extracellular proteins (**a**), polysaccharides concentration (**b**) and correlation analysis between flocculating ability and extracellular proteins (**c**) of *Chlorococcum* sp. GD under different ammonia nitrogen concentrations.

In the present study, the concentration of proteins and polysaccharides from EPS attached to the cell surface of *Chlorococcum* sp. GD cultivated in different ammonia nitrogen concentrations is measured (Figure 2a,b). The proteins concentration significantly increased from 31.4 mg·g·DW^−1^ to 83.9, 67.3, 81.5, and 79 mg·g·DW^−1^, respectively, after 4 days of cultivation of *Chlorococcum* sp. GD in wastewater with 10, 20, 30, and 50 mg·L^−1^ ammonia nitrogen. The proteins concentration then decreased to 28.6, 38.8, 43.9, and 35.1 mg·g·DW^−1^, respectively, at the end of cultivation. The polysaccharides concentration of *Chlorococcum* sp. GD in wastewater with 10 and 20 mg·L^−1^ ammonia nitrogen slightly increased from 21.7 mg·g·DW^−1^ to 26 and 24 mg·g·DW^−1^, respectively, after 4 days of cultivation. It then decreased significantly to 6.0 and 7.8 mg·g·DW^−1^, respectively, at the end of cultivation. The polysaccharides concentration of *Chlorococcum* sp. GD in wastewater with 30 and 50 mg·L^−1^ ammonia nitrogen significantly decreased from 21.7 mg·g·DW^−1^ to 5.4 and 5.9 mg·g·DW^−1^, respectively, throughout the culture period. Based on data above, it indicated that both proteins and polysaccharides content were dynamic regardless of ammonia nitrogen concentrations in wastewater throughout the culture period. It was obvious that the proteins content from EPS was higher than the polysaccharides content at each growth stage of *Chlorococcum* sp. GD, and the peak of extracellular protein content of *Chlorococcum* sp. GD appeared in day 4 for all treatments. It was also found that *Chlorococcum* sp. GD in wastewater with 10 mg·L^−1^ ammonia nitrogen had the lowest concentration of proteins at the end of the culture.

As shown in Figure 2c, the relationship between extracellular protein content and flocculation efficiency in each treatment is analyzed. When the flocculation efficiency was compared with the proteins content, it was seen that there was a definite trend. In practice, there was a good degree of correlation (*r* = 0.9, *p* < 0.05) when *Chlorococcum* sp. GD was cultivated in wastewater with 20 mg·L^−1^ ammonia nitrogen. That is to say, flocculation might be positively related to extracellular protein content in EPS.

### 3.2. The Growth and Biomass Production of Chlorococcum sp. GD in Wastewater with Different Ammonia Nitrogen Concentrations

**Figure 3:**
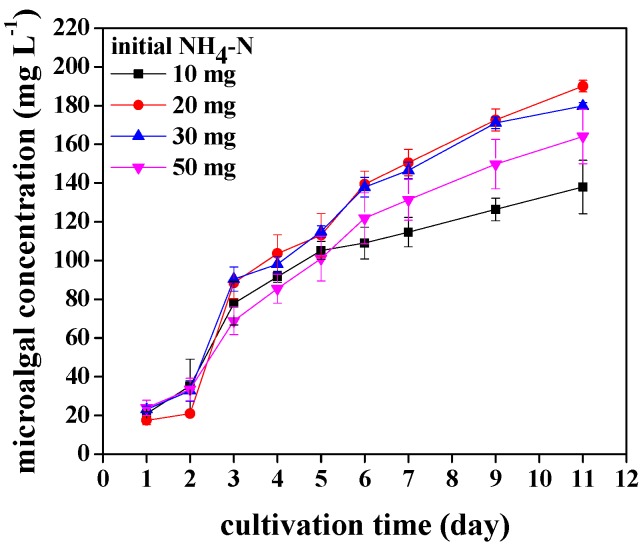
Biomass production of *Chlorococcum* sp. GD under different ammonia nitrogen concentrations.

The growth potential of *Chlorococcum* sp. GD with different ammonia nitrogen concentrations is shown in Figure 3. There was no lag phase of growth curves, which indicated that *Chlorococcum* sp. GD could well adapt in synthetic wastewater with different ammonia nitrogen concentrations. After 11 days of cultivation, the biomass production increased and ranged from 138 to 190.1 mg·L^−1^. The biomass production was highest when ammonia nitrogen concentration in synthetic municipal wastewater was 20 mg·L^−1^. The specific growth rate of *Chlorococcum* sp. GD cultivated in wastewater with different ammonia nitrogen concentrations is also calculated as shown in Table 1. The specific growth rate was significantly affected by ammonia nitrogen concentration (*p* < 0.05). *Chlorococcum* sp. GD cultivated in 20 mg·L^−1^ ammonia nitrogen had higher specific growth rate (0.24 d^−1^) than other ammonia nitrogen concentrations (0.19–0.21 d^−1^) (Table 1). The mean biomass productivity was also the highest when *Chlorococcum* sp. GD was cultivated in 20 mg·L^−1^ ammonia nitrogen. Similarly, when *Chlorococcum* sp. GD grew in wastewater with 20 mg·L^−1^ ammonia nitrogen, the double time was the shortest (Table 1). All results above demonstrated that the growth and biomass production of *Chlorococcum* sp. GD were significantly affected by ammonia nitrogen, and the most suitable ammonia nitrogen concentration for the growth of *Chlorococcum* sp. GD was 20 mg·L^−1^.

### 3.3. Pollutants Removal of Chlorococcum sp. GD in Wastewater with Different Ammonia Nitrogen Concentrations

**Figure 4:**
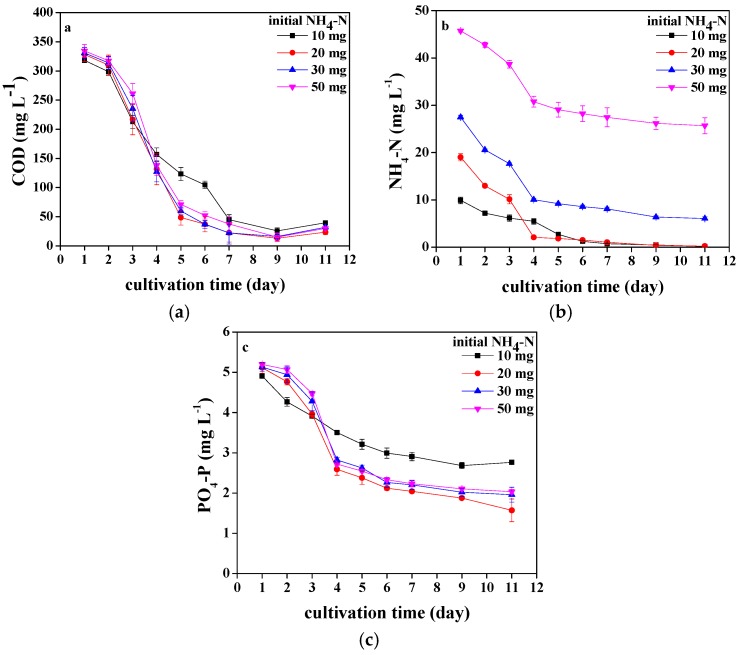
COD (**a**), ammonia nitrogen (**b**), and phosphorous (**c**) removal of *Chlorococcum* sp. GD under different ammonia nitrogen concentrations.

As shown in Figure 4a, the organic pollutant concentration decreased at the end of the culture in every ammonia nitrogen concentration group. The COD removal efficiencies and rates varied from 87.6% to 92.8% and from 25.3 to 27.7 mg COD·L^−1^·d^−1^, respectively. *Chlorococcum* sp. GD showed excellent COD removal performance, and COD removal did not seem to be affected by ammonia nitrogen concentration. Conversely, the ammonia nitrogen removal efficiency was related to the ammonia nitrogen concentration. The ammonia nitrogen removal efficiency was around 98% when the ammonia nitrogen concentration of wastewater was 10 and 20 mg·L^−^^1^. The removal efficiency decreased to 43.9–78.0% when the initial ammonia nitrogen concentration was more than 20 mg·L^−1^ (Figure 4b). Although the phosphorus removal efficiency was less than 70% in every ammonia nitrogen concentration group (Figure 4c), the phosphorus removal efficiency under 20–50 mg·L^−1^ ammonia nitrogen was superior to that under 10 mg·L^−1^ ammonia nitrogen. According to the above results, pollutants removal performance of *Chlorococcum* sp. GD was significantly affected by ammonia nitrogen concentration, and pollutants removal efficiency was greatest when the ammonia nitrogen concentration was 20 mg·L^−1^. It was also found that most of pollutants were removed from wastewater after 4–5 days of cultivation of *Chlorococcum* sp. GD in each treatment.

**Figure 5:**
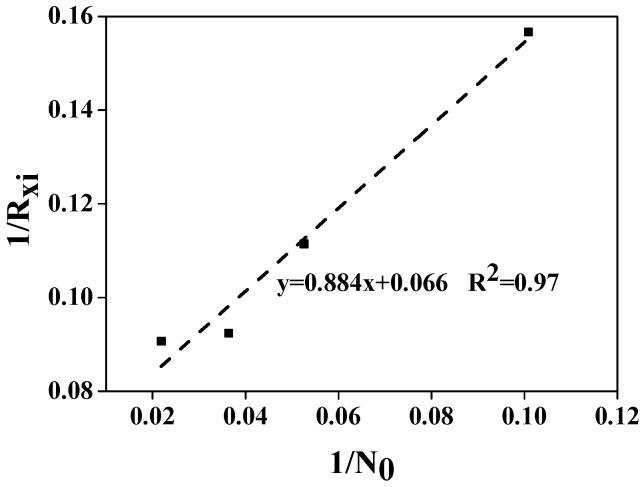
Determination of kinetic coefficients V_max_ and K_m_ for NH_4_-N removal of *Chlorococcum* sp. GD.

The data on ammonia nitrogen removal of *Chlorococcum* sp. GD given in Figure 4b is plotted in form of 1/$R_{X_{i}}$ versus 1/(NH_4_-N)_0_ as shown in Figure 5. From the slope and intercept of best fit line of this plot, kinetic coefficients of ammonia nitrogen removal by *Chlorococcum* sp. GD were V_max_ = 15.2 mg NH_4_-N·g^−1^ DW·d^−1^ and K_m_ = 13.4 mg NH_4_-N·L^−1^.

### 3.4. Lipid Accumulation of Chlorococcum sp. GD in Wastewater with Different Ammonia Nitrogen Concentrations

**Figure 6:**
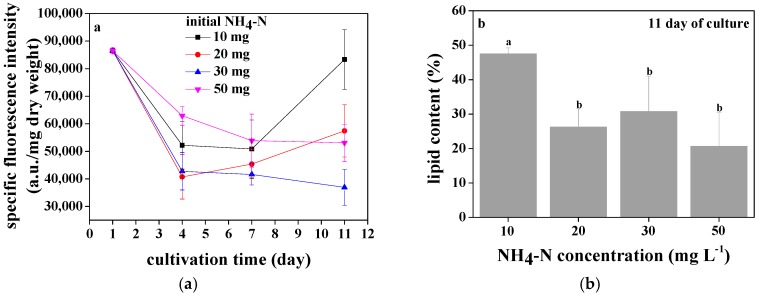
The specific fluorescence intensity (**a**) and lipid content (**b**) of *Chlorococcum* sp. GD under different ammonia nitrogen concentrations. In (**b**), different letters on columns indicated that they were significantly different at a probability level of 0.05 according to ANOVA test.

The lipid accumulation of *Chlorococcum* sp. GD cultivated with different ammonia nitrogen concentrations is shown in Figure 6a. The lipid accumulation was affected by the initial ammonia nitrogen concentration. When *Chlorococcum* sp. GD was cultivated with 10 and 20 mg·L^−1^ ammonia nitrogen, the lipid accumulation firstly decreased and then increased. The difference was that the turning point for *Chlorococcum* sp. GD cultivated with 10 mg·L^−1^ ammonia nitrogen was in the 7th day, whereas the turning point for *Chlorococcum* sp. GD cultivated with 20 mg·L^−1^ ammonia nitrogen was in the 4th day. When *Chlorococcum* sp. GD was cultivated with 30 and 50 mg·L^−1^ ammonia nitrogen, the lipid accumulation decreased during the whole culture period. In conclusion, after 11 days of culture, the highest lipid accumulation of *Chlorococcum* sp. GD occurred when cultivated with 10 mg·L^−1^ ammonia nitrogen, followed by 20 and 50 mg·L^−1^ ammonia nitrogen. *Chlorococcum* sp. GD had lowest lipid accumulation when cultivated with 30 mg·L^−1^ ammonia nitrogen. The lipid content of *Chlorococcum* sp. GD cultivated with different ammonia nitrogen concentrations is shown in Figure 6b, according to the gravimetric method. After 11 days of culture with wastewater of 10 mg·L^−1^ ammonia nitrogen, *Chlorococcum* sp. GD had the highest lipid content at 47.5%, which was significantly higher than that of *Chlorococcum* sp. GD cultivated with wastewater of 20, 30, and 50 mg·L^−1^ ammonia nitrogen (*p* < 0.05). The lipid content of *Chlorococcum* sp. GD cultivated with 20, 30, and 50 mg·L^−1^ ammonia nitrogen was of the same order of magnitude.

## 4. Discussion

In the present study, *Chlorococcum* sp. GD cultivated in wastewater with high ammonia nitrogen concentration (20–50 mg·L^−1^) had high concentration of extracellular proteins at the end of the culture. Nitrogen is an essential nutrient that is required in microalgal growth and is used to synthesize a variety of biological substances, such as peptides, proteins, etc. [29]. Durmaz and Sanin [30] found that EPS of activated sludge had high protein and low carbohydrate content at a C/N ratio of 5. When the C/N ratio increased to 40, the proteins content decreased sharply, whereas the carbohydrates content increased. Thus, it could be seen that nitrogen deficiency in high C/N ratio was not beneficial to the synthesis of extracellular proteins, which was reconfirmed in this study. It was also found that there was a positive trend between flocculation efficiency of *Chlorococcum* sp. GD and protein content. Salim et al. [15] found that autoflocculation of *E. texensis* was due to extracellular proteins patched to the cell surface. As reported by Díaz-Santos et al. [31], a soluble cell wall protein similar to glucanases was isolated from the yeast *Saccharomyces bayanus* var. *uvarum* and could effectively induce flocculation of *Chlamydomonas reinhardtii* and *Picochlorum* sp. HM1. Díaz-Santos et al. [32] expressed the *FLO5* gene (flocculation gene, specific cell surface lectin-like glycoproteins relating to the flocculation process in yeasts) from *S*. *bayanus* by cotransformation in *C*. *reinhardtii*. *C*. *reinhardtii* transformants exhibited self-flocculation abilities between 2- and 3.5-fold higher than the control untransformed strain. All these cases demonstrated the importance of extracellular proteins in microalgal flocculation, which was proved again in this study. Based on the analysis and discussion above, it was speculated that nitrogen indeed affected the flocculation efficiency of *Chlorococcum* sp. GD, which was likely related to the production of extracellular proteins. It was found that the flocculating ability of *Chlorococcum* sp. GD was affected by ammonia nitrogen. *Chlorococcum* sp. GD had an excellent flocculating performance when ammonia nitrogen concentration of wastewater was more than 10 mg·L^−1^. *Chlorococcum* sp. GD had the highest flocculating efficiency in the middle of the culture (4–5 days) regardless of initial ammonia nitrogen concentration in wastewater. The phenomenon likely came from the maximum extracellular protein content and high degradation efficiency of pollutants of *Chlorococcum* sp. GD synchronously in the middle of the culture (4–5 days). Of course, the extracellular proteins content of *Chlorococcum* sp. GD was obviously higher than the extracellular polysaccharides content, which was similar to self-flocculating microalga *E*. *texensis,* since it had high extracellular proteins content [12,15].

It was found that ammonia nitrogen affected the growth of *Chlorococcum* sp. GD. The optimal ammonia nitrogen concentration for its growth was 20 mg·L^−1^, which was consistent with when Tam and Wong [18] showed that *C*. *vulgaris* grew well in cultures containing 20 mg·L^−1^ ammonia nitrogen. Of course, many microalgae could grow well in cultures with more than 20 mg·L^−1^ ammonia nitrogen [19,33], which might be species-dependent or related to other factors, such as light intensity, immobilization, operation modes, etc. [34,35]. In this study, the 190.1 mg·L^−1^ of microalgal biomass was low compared to the common biomass levels of *Chlorococcum* sp. in BG11 (360–4140 mg·L^−1^) [36]. The possible reason was the low initial inoculation concentration used in the study.

The pollutants removal was also affected by ammonia nitrogen, and pollutants removal efficiency was the best when the ammonia nitrogen concentration was 20 mg·L^−1^. Commonly, the elementary composition ratio of microalgal cells gave a hint of the optimal N/P ratio in wastewaters. The empirical elementary composition for microalgae was C_106_H_263_O_110_N_16_P (N/P ratio = 7.2:1). In our work, the optimal N/P ratio for pollutants removal was 4:1, which was relatively lower than the empirical value. Some investigations also found that *Klebsormidium* sp. and *Pseudanabaena* sp. had excellent nitrogen and phosphorus removal capability with high N/P ratio of 7–10 and 7–20 [37], which was higher than the empirical value. Therefore, the optimal N/P ratio for pollutants removal is likely to depend on the strain and growth conditions. Kinetics of ammonia nitrogen removal was calculated by Michaelis-Menten Kinetics. Kinetic coefficients of ammonia nitrogen removal by *Chlorococcum* sp. GD were V_max_ = 15.2 mg NH_4_-N·g^−1^ DW·d^−1^ and K_m_ = 13.4 mg NH_4_-N·L^−1^, which was lower than those of *Klebsormidium* sp. and *Pseudanabaena* sp. [37]. Normally, a high V_max_ is an indicator of the high potential capability of removing pollutants, while a low K_m_ meant that the microalga could reach its highest pollutant removal rate at low pollutant concentrations [37]. Based on the analysis above, it is indicated that *Chlorococcum* sp. GD has a competitive advantage in wastewaters with relatively low ammonia nitrogen concentration.

In this work, it was found that the lipid content of *Chlorococcum* sp. GD was influenced by ammonia nitrogen. Nitrogen deprivation was commonly the main trigger for lipid accumulation of microalgae [38]. Because of nitrogen deprivation of initial inoculum cultivated in BG11 for 13 days, it had the highest lipid content. For *Chlorococcum* sp. GD cultivated with 30 and 50 mg·L^−1^ ammonia nitrogen, there was still a large amount of nitrogen not being degraded at the end of the culture. Microalgal cells consequently did not accumulate large amounts of lipids, and the lipid content was relatively low. For low nitrogen concentration groups (10 and 20 mg·L^−1^), almost all ammonia nitrogen was degraded within 7 days of culture. Afterwards, *Chlorococcum* sp. GD was in the stage of nitrogen deprivation, and thus accumulated high content of lipid. Of course, the lipid content in 10 mg·L^−1^ nitrogen concentration group was significantly higher than that in 20 mg·L^−1^ nitrogen concentration group. Nevertheless, the total lipid content in 20 mg·L^−1^ nitrogen concentration group (10,913,050 a.u. L^−1^) was almost the same as that in 10 mg·L^−1^ nitrogen concentration group (11,498,226.9 a.u. L^−1^) owing to high biomass production of *Chlorococcum* sp. GD cultivated with 20 mg·L^−1^ ammonia nitrogen. High pollutants removal efficiency also occurred in wastewater with 20 mg·L^−1^ ammonia nitrogen. Therefore, it was suggested that *Chlorococcum* sp. GD was cultivated with 20 mg·L^−1^ ammonia nitrogen for lipid production. In the study, 26.3% of lipid content was achieved when *Chlorococcum* sp. GD was cultured with 20 mg·L^−1^ ammonia nitrogen, which was comparable to lipid content of microalgae cultivated in wastewaters in former literatures [39,40]. Interestingly, for the cultivation of *Chlorococcum* sp. GD, high efficient accumulation of lipid occurred at the end of the culture, while optimum efficiency of pollutants degradation and microalgal harvesting occurred in the middle of the culture. Therefore, it was worth paying attention to how to solve this contradiction in the future.

## 5. Conclusions

In the study, it was found that the flocculating ability of *Chlorococcum* sp. GD was affected by ammonia nitrogen concentration in wastewater. Relative high ammonia nitrogen concentration (20–50 mg·L^−1^) was beneficial to flocculation, and the highest flocculating efficiency was in the middle of the culture (4–5 days) regardless of ammonia nitrogen concentration in wastewater. It was speculated that high flocculating efficiency was likely related to the production of extracellular proteins. Relative high ammonia nitrogen concentration (20 mg·L^−1^) was also beneficial to biomass production and lipid accumulation of *Chlorococcum* sp. GD. Pollutants removal efficiency was the best when the ammonia nitrogen concentration was 20 mg·L^−1^. A better understanding of the response of *Chlorococcum* sp. GD to ammonia nitrogen is beneficial for cultivating and harvesting self-flocculating microalga from wastewater.

## Figures and Tables

**Table 1:** Growth parameters of *Chlorococcum* sp. GD under different ammonia nitrogen concentrations.

Ammonia Concentration (mg·L^−1^)	N/P Ratio	Specific Growth Rate (μ, d^−1^)	Mean Biomass Productivity (mg DW·L^−1^·d^−1^)	Double Time (d^−1^)
10	2	0.19 ± 0.02 ^a^	10.64 ± 1.35 ^a^	2.37 ± 0.08 ^a^
20	4	0.24 ± 0.01 ^b^	15.70 ± 0.48 ^b^	2.12 ± 0.06 ^b^
30	6	0.21 ± 0.01 ^a^	14.26 ± 0.06 ^b,c^	2.27 ± 0.03 ^a,b^
50	10	0.19 ± 0.03 ^a^	12.74 ± 1.61 ^c^	2.34 ± 0.1 ^a^

Different letters (a, b and c) followed by values on columns indicated that they were significantly different at a probability level of 0.05 according to ANOVA test. DW: dry weight.
